# scMUSCL: multi-source transfer learning for clustering scRNA-seq data

**DOI:** 10.1093/bioinformatics/btaf137

**Published:** 2025-03-27

**Authors:** Arash Khoeini, Funda Sar, Yen-Yi Lin, Colin Collins, Martin Ester

**Affiliations:** School of Computing Science, Simon Fraser University, Burnaby, British Columbia V5A 1S6, Canada; Vancouver Prostate Centre, Vancouver, British Columbia V6H 3Z6, Canada; Vancouver Prostate Centre, Vancouver, British Columbia V6H 3Z6, Canada; Department of Urologic Science, University of British Columbia, Vancouver, British Columbia V5Z 1M9, Canada; Vancouver Prostate Centre, Vancouver, British Columbia V6H 3Z6, Canada; Department of Urologic Science, University of British Columbia, Vancouver, British Columbia V5Z 1M9, Canada; School of Computing Science, Simon Fraser University, Burnaby, British Columbia V5A 1S6, Canada; Vancouver Prostate Centre, Vancouver, British Columbia V6H 3Z6, Canada

## Abstract

**Motivation:**

Single-cell RNA sequencing (scRNA-seq) analysis relies heavily on effective clustering to facilitate numerous downstream applications. Although several machine learning methods have been developed to enhance single-cell clustering, most are fully unsupervised and overlook the rich repository of annotated datasets available from previous single-cell experiments. Since cells are inherently high-dimensional entities, unsupervised clustering can often result in clusters that lack biological relevance. Leveraging annotated scRNA-seq datasets as a reference can significantly enhance clustering performance, enabling the identification of biologically meaningful clusters in target datasets.

**Results:**

In this article, we propose Single Cell MUlti-Source CLustering (scMUSCL), a novel transfer learning method designed to identify cell clusters in a target dataset by leveraging knowledge from multiple annotated reference datasets. scMUSCL employs a deep neural network to extract domain- and batch-invariant cell representations, effectively addressing discrepancies across various source datasets and between source and target datasets within the new representation space. Unlike existing methods, scMUSCL does not require prior knowledge of the number of clusters in the target dataset and eliminates the need for batch correction between source and target datasets. We conduct extensive experiments using 20 real-life datasets, demonstrating that scMUSCL consistently outperforms existing unsupervised and transfer learning-based methods. Furthermore, our experiments show that scMUSCL benefits from multiple source datasets as learning references and accurately estimates the number of clusters.

**Availability and implementation:**

The Python implementation of scMUSCL is available at https://github.com/arashkhoeini/scMUSCL.

## 1 Introduction

Sequence analysis is crucial in molecular biology due to the sequential structure of genomes and proteins. It encompasses various tasks, such as detecting mutations and measuring gene expression levels. Gene expression, indicated by the number of gene transcripts that may translate into proteins, provides insights into biological processes and cellular functions. Traditional sequence analysis technologies yield aggregate measurements, like the average gene expression level across a sample, thus losing transcriptional heterogeneity. Recently, single-cell RNA sequencing (scRNA-seq) has emerged, offering gene expression profiles at the resolution of individual cells, thereby preserving this heterogeneity and providing finer insights into cellular functions.

While scRNA-seq data provide information at a great level of resolution, it comes with the following two challenges. First, scRNA-seq data are very high-dimensional, since the number of genes is in the order of tens of thousands, i.e. roughly 30 000 for humans. Consequently, methods for the analysis of scRNA-seq data typically perform dimensionality reduction. Second, scRNA-seq data are very noisy. Several factors contribute to the noise, including the stochasticity of the transcription, technical factors, such as mRNA capture rate and low accuracy in measuring genes that are expressed only at a low level. Clustering cells alleviates the effect of the noise and is an essential step before downstream tasks, i.e. cell-type annotation, visualization, trajectory analysis, and determination of transcriptional heterogeneity in health and disease (Luecken and Theis 2019, Wang *et al.* 2019, Clarke *et al.* 2021).

Clustering is an unsupervised task that identifies groups whose members are more similar to each other than to members of other groups. Numerous methods have been proposed in the literature to identify cell clusters or to derive low-dimensional representations that facilitate clustering (Wang *et al.* 2018, Tian *et al.* 2019, Ciortan and Defrance 2021, Wan *et al.* 2022). A common limitation in almost all of these methods is that they are completely unsupervised and do not take advantage of the wealth of information available in publicly annotated scRNA-seq datasets (Grabski *et al.* 2023, Zhang *et al.* 2023).

**Figure 1.**
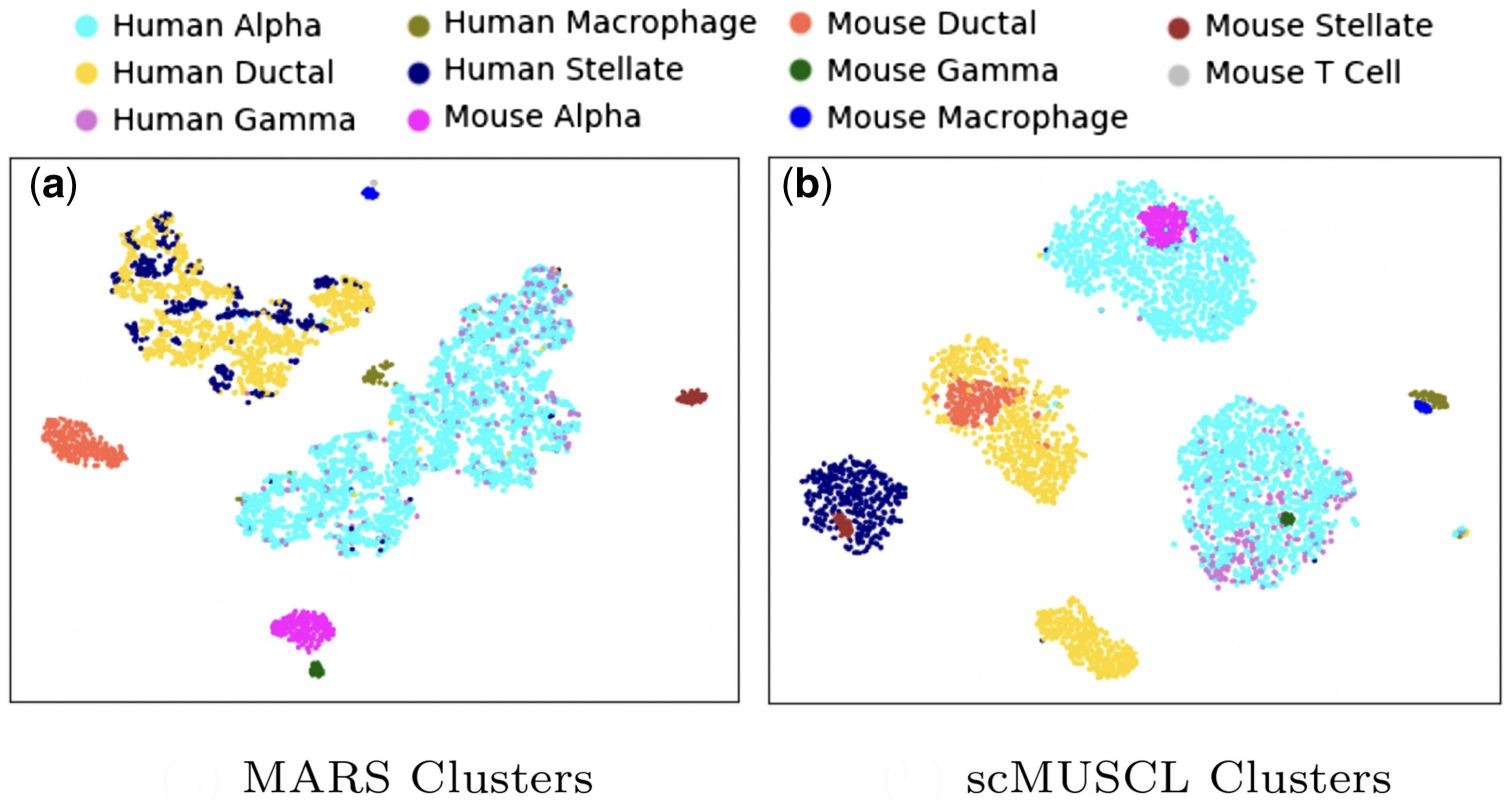
TSNE plot of cells in a mouse and a human pancreas cells when a mouse tissue is used as the source dataset to cluster human cells. (a) The clusters generated by MARS and (b) scMUSCL’s clustering result. This illustrates how MARS fails in aligning human and mouse cells. For example, MARS creates a cluster for mouse alpha cells far away from human alpha cells, while scMUSCL successfully aligns these two clusters. More interestingly, we can see that scMUSCL aligns rare cell-types, such as human and mouse macrophage cells, while MARS fails to do so.

Transfer learning seeks to improve the performance of a machine learning method in a target domain by leveraging relevant knowledge from different, but related, source domains (Zhuang *et al.* 2021). Using available annotated data can guide a clustering algorithm in identifying more accurate and biologically meaningful clusters, such as those corresponding to specific cell types. However, incorporating existing annotated scRNA-seq data into a clustering algorithm is not straightforward. A major challenge in leveraging available scRNA-seq datasets is the discrepancies that exist between datasets from different species, different tissues of the same species, or different sequencing technologies and laboratories. For example, MARS (Brbić *et al.* 2020) aims to leverage multiple annotated scRNA-seq datasets as source domains to learn a clustering for an unannotated target domain. However, MARS does not explicitly align the source datasets with each other or with the target dataset, leading to distribution discrepancies (e.g. batch effects) between the source and target datasets (illustrated in Fig. 1a). These discrepancies limit MARS’s ability to learn from common source clusters and distinguish them from new clusters that appear only in the target domain. Additionally, most previous work on cell clustering assumes the true number of clusters in advance, which is a significant drawback, since, in practice, the actual number of clusters is unknown.

In this article, we introduce Single Cell MUlti-Source CLustering (scMUSCL), a novel multi-source transfer learning method for clustering scRNA-seq data. scMUSCL leverages multiple annotated source scRNA-seq datasets to learn the characteristics of different cell types and transfers this knowledge to identify cell clusters in a target dataset. scMUSCL can automatically determine the number of clusters and it is composed of three stages. The first stage involves the contrastive pre-training of the feature extractor, which initializes a feature space that maps similar cells closer together. In the second stage, cluster initialization, we create two sets of clusters: one for all combined source datasets and another for the target dataset. The final stage, fine-tuning, addresses distribution discrepancies and mitigates batch effects by enabling scMUSCL to perform cell-level and cluster-level alignment. In this stage, scMUSCL learns domain-invariant feature representations necessary for transferring knowledge from the source domains to the target domain. During fine-tuning, scMUSCL further leverages the source datasets to learn a clustering for the target dataset. The fine-tuning process is guided by a loss function with two terms. The first term performs cell-level alignment by iteratively aligning cells of common cell types across different source datasets and forming compact clusters in both the source and target domains. The second term performs iterative cluster-level alignment, aligning source and target clusters of common cell types, and prevents the feature extractor from learning collapsing solutions. Extensive experiments on 20 real-world human and mouse tissue datasets demonstrate the superiority of scMUSCL over state-of-the-art scRNA-seq clustering methods. The main contributions of this article are as follows:

- We introduce scMUSCL, a novel multi-source transfer learning framework specifically designed for clustering scRNA-seq data.
- Through extensive experiments on 20 real-world datasets, we demonstrate that scMUSCL effectively transfers knowledge across diverse biological contexts, including different species, platforms, and tissues. Our method consistently outperforms previous scRNA-seq clustering approaches in terms of clustering accuracy and robustness.
- We highlight the benefits of leveraging multiple source datasets as learning references, showing that scMUSCL accurately estimates the number of clusters even in challenging scenarios with complex biological variability.
- We provide evidence of the scalability of scMUSCL, demonstrating its linear runtime and memory efficiency with respect to the number of source cells, target cells, and genes making it suitable for large-scale scRNA-seq studies.

## 2 The scMUSCL method

We start this section with the problem definition and then we give an overview of scMUSCL, a transfer-learning-based clustering method to find cell clusters using scRNA-seq data.

We are given |S| labeled source datasets $D^{s_{l}}=\{X^{s_{l}},y^{s_{l}}\}$ for 1≤l≤|S| where $X^{s_{l}}\in R^{|D^{s_{l}}|\times v}$ is a matrix containing $\left|D^{s_{l}}\right|$  v-dimensional cells. v is the size of the input feature space, or here the number of genes. $y^{s_{l}}$ is a vector such that $y_{i}^{s_{l}}$ indicates the annotation (e.g. cell-type) for the ith cell in the source dataset $D^{s_{l}}$. We are also given an unlabeled target dataset $D^{t}=\{X^{t}\}$. Our goal is to cluster cells in $D^{t}$, such that each cluster represents either a seen or unseen cell-type. An unseen cell-type is a cell-type that exists in $D^{t}$ but not in $D^{s_{l}}$ for 1≤l≤|S|.

scMUSCL consists of a feature extractor G, which is a deep neural network that maps cells into a d-dimensional latent feature space where d≪v. scMUSCL leverages multiple annotated scRNA-seq datasets as source data and a single unannotated scRNA-seq experiment as the target dataset for clustering. The method learns the clustering of the target dataset through a three-stage process: contrastive pre-training, cluster initialization, and fine-tuning. Each stage is detailed below.

### 2.1 Contrastive pre-training

In the first stage, we pre-train the feature extractor *G* using both the target and source datasets. During pre-training, we employ the InfoNCE contrastive loss (Oord *et al.* 2018). Specifically, we randomly sample a minibatch of N cells and form positive pairs, where each pair consists of a cell from the minibatch and its augmented version, resulting in a total of 2 *N* cells. To generate the augmented version of a cell, we randomly select non-zero gene expressions and set them to zero with a probability *P*. The InfoNCE loss for a positive pair of examples (i,j) is defined as:

(1) $$l_{i,j}=-\text{log}\;\frac{\;\text{exp}(\mathrm{sim}(z_{i},z_{j})/\tau )}{\sum_{k=1}^{2N}1_{\left\{k\neq i\right\}}\;\text{exp}(\mathrm{sim}(z_{i},z_{k})/\tau )},$$

where 1 denotes the indicator function, sim is a similarity function, and τ is a temperature parameter which we set to 1. Here we define the similarity function as the dot product of two vectors: $\mathrm{sim}(u,v)=u^{T}v$. We did not use a projection head (Chen *et al.* 2020) and z is the direct output of our feature extractor G. The final pre-training loss is computed across all positive pairs, both (i,j) and (j,i), in a mini-batch with size N.

### 2.2 Cluster initialization

**Figure 2.**
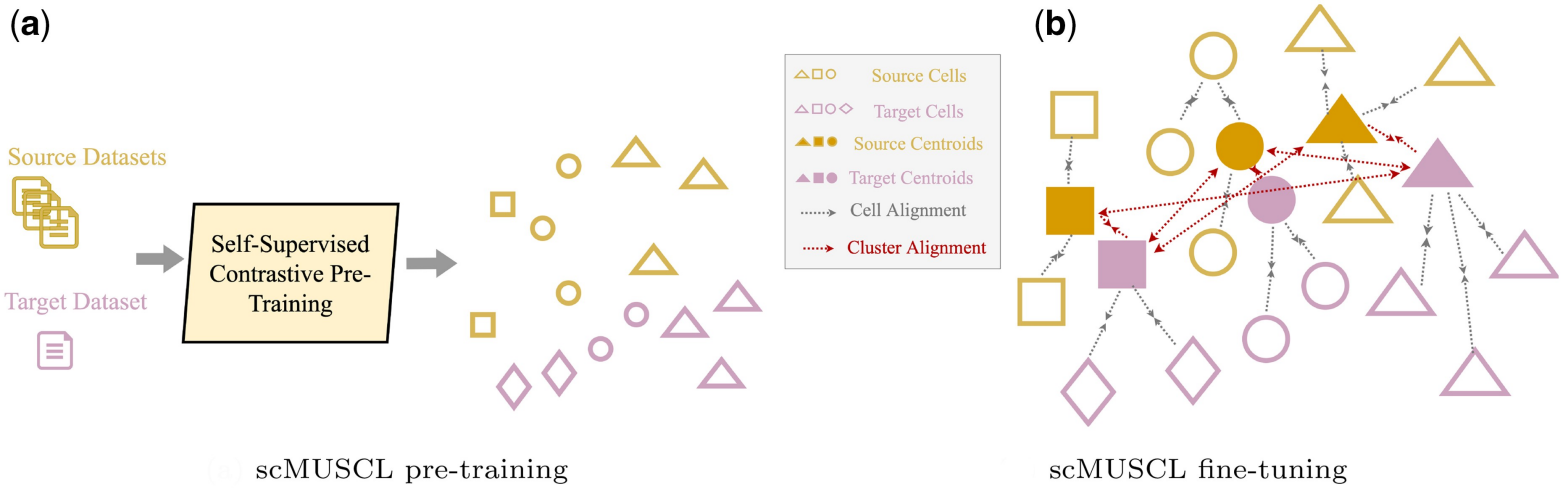
Illustration of scMUSCL’s workflow. Each shape represents a cell-type and filled shapes are cluster representatives. (a) Pre-training: Source and target datasets are combined to train a neural network encoder using self-supervised contrastive learning. This step learns a shared representation space where clusters corresponding to different cell types (shapes) are dispersed. (b) Fine-tuning: Cluster representatives (filled shapes) are initialized by averaging source cells of each cell type. During fine-tuning, cell alignment loss aligns individual cells with the same cell type across all source datasets, creating compact clusters in both source and target domains. Simultaneously, cluster alignment loss adjusts cluster representatives by minimizing the distance between representatives of the same cell type across domains while repelling other clusters.

In the second stage of our method, we utilize the pre-trained feature extractor G from the first stage to compute latent representations for all cells of the source datasets. These representations are used to initialize $C^{s}$, which is the set of cluster centroids for the combined source datasets, residing in $R^{n\times d}$. Here, n denotes the number of unique cell types across all source datasets. We define each row k in $C^{s}$ as $C_{k}^{s}$ and express it as follows:

(2) $$C_{k}^{s}=\frac{1}{n_{k}^{s}}\sum_{l=1}^{\left|S\right|}\sum_{i=1}^{\left|D^{s_{l}}\right|}1_{\left\{y_{i}^{s_{l}}=k\right\}}G(X_{i}^{s_{l}}),$$

where $n_{k}^{s}=\sum_{l=1}^{\left|S\right|}\sum_{i=1}^{\left|D^{s_{l}}\right|}1_{\left\{y_{i}^{s_{l}}=k\right\}}$ signifies the total number of cells in cell-type k across all source datasets. Following the initialization of $C^{s}$, we duplicate its values to establish the initial values for the target cluster representatives, $C^{t}$, such that $C^{t}=C^{s}$ at this stage. This approach hinges on the hypothesis that the pre-training phase creates a space where cells of the same type from both source and target datasets are closer to each other than cells of different types. Essentially, if two groups of cells—one from the source and another from the target—share the same cell type, they should be proximate in this representation space (illustrated in Fig. 2a). Accordingly, initializing two cluster centroids in this region—one for the source and one for the target—makes logical sense. However, we acknowledge that the target dataset may include cells with cell types that were not observed in the source datasets. Consequently, not all target clusters will necessarily correspond to clusters in the source datasets. These cells with unseen cell types will initially align with the nearest target cluster centroid and gradually shift that centroid towards themselves during the fine-tuning stage. Initially, the number of target clusters in scMUSCL is aligned with the number of clusters from the source datasets. However, because the target dataset combines data from multiple sources, it often contains fewer distinct cell types compared to the combined sources. This initialization results in an excess of target cluster representatives. During the fine-tuning process, each cell in the target dataset aligns with its nearest cluster representative, gradually shifting centroids closer to the cells they represent. If any centroid in $C^{t}$ fails to attract any cells during this process, it remains unused. This adaptive mechanism allows scMUSCL to effectively determine the optimal number of clusters required to accurately represent the cell types present in the target dataset.

**Figure 3.**
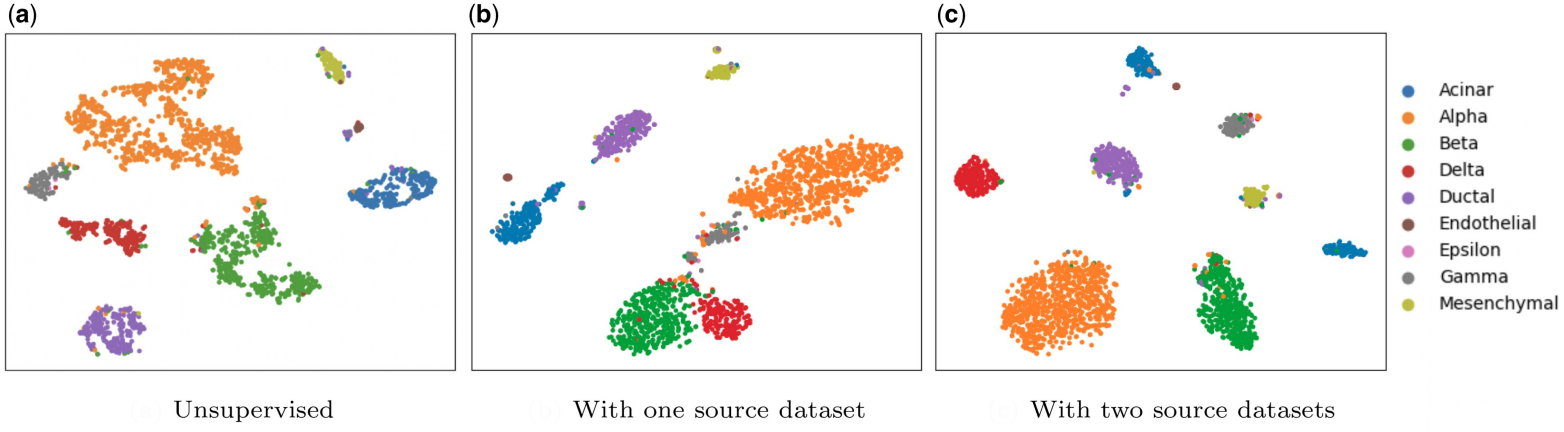
TSNE plots of cells in a human pancreas tissue (Murano). (a) Cell clusters when we use no source dataset as the reference. We only use contrastive learning to learn the latent representations, silhouette score = 0.03. In (b) we used one mouse pancreas tissue (Baron) as the source dataset, silhouette score = 0.76, and in (c), we used two mouse pancreas source datasets (Baron and Tabula Muris), silhouette score = 0.78.

### 2.3 Fine-tuning

In the last stage we fine-tune our feature extractor and optimize cluster representatives. We aim for four different goals in the fine-tuning stage:

1. To align cells with the same cell-type across different source datasets. Different source datasets may have cells with the same cell type, but their expression profiles may exhibit domain shift (e.g. batch effect). It is necessary to align these cells because otherwise the feature extractor might learn domain discriminative features, i.e. features that carry information about the domain rather than about the cell-type (Stojanov *et al.* 2021).
2. To learn compact target clusters. We seek to find compact clusters such that cells with the same cell-type are much closer to each other than cells with different cell-types.
3. To align source and target clusters of common cell-types. We assume the source datasets are highly relevant to the target dataset, which means they share some cell-types. We want to align these clusters with common cell-types in order to effectively transfer extracted knowledge from source datasets to the target dataset.
4. To learn well-separated clusters. We seek to find a representation space in which cells with different cell-types are located far apart.

Hence, we propose a loss function with two terms. The first loss term, cell alignment loss, aims at the first two above goals. The second loss term, cluster alignment loss, aims at the latter two goals. In the following subsections we explain these two terms in detail.

#### 2.3.1 Cell alignment loss

The cell alignment loss term minimizes the distance between the latent representation $G(X_{i}^{s_{l}})$ of the *i*th cell from the source dataset $D^{s_{l}}$ and its corresponding cluster centroid. Given that source datasets are annotated, each cell $X_{i}^{s_{l}}$ has a known gold standard cell type $y_{i}^{s_{l}}$. Since source clusters are consistent across all datasets, minimizing the distance between a cell and its cluster centroid ensures alignment at the cell-type level across source datasets. This alignment not only facilitates scMUSCL in learning compact clusters for the source datasets but also enhances its ability to generalize effectively. The cell alignment loss is depicted in Fig. 2b and is computed separately for each source dataset as specified below:

(3) $$\begin{array}{c} L_{\text{cell}}^{s_{l}}=\frac{1}{(|S|+1)K}\sum_{k=1}^{K}\frac{1}{\sum_{i=1}^{\left|D^{s_{l}}\right|}1_{\left\{y_{i}^{s_{l}}=k\right\}}}\\ \sum_{i=1}^{\left|D^{s_{l}}\right|}1_{\left\{y_{i}^{s_{l}}=k\right\}}d\mathrm{ist}(G(X_{i}^{s_{l}}),C_{k}^{s}) \end{array}.$$

In our implementation, we utilized the Euclidean distance for dist(.), though any distance function could be substituted. The coefficient $\frac{1}{|S|+1}$ is employed to evenly weight all source and target datasets.

For the target dataset, the loss function follows a similar principle, but with a distinction: we aim to minimize the distance between the latent features of an unannotated cell and its nearest cluster centroid, as the gold standard cell-type is unknown for these cells. We define the loss term for target cell alignment as below:

(4) $$L_{\mathrm{cell}}^{t}=\frac{1}{(|S|+1)K}\sum_{k=1}^{K}\frac{1}{\left|N_{k}^{t}\right|}\sum_{i\in N_{k}^{t}}1_{\left\{y_{i}^{t}=k\right\}}d\mathrm{ist}(G(X_{i}^{t}),C_{k}^{t}),$$

where $N_{k}^{t}$ is the set of target cells that their nearest target cluster representative is k.

#### 2.3.2 Cluster alignment loss

Enforcing cluster compactness is desirable, but it is also the recipe for collapse, as the feature extractor might minimize the loss by mapping all the cells and cluster representatives into one vector. Therefore, we need to ensure clusters are well-separated to avoid collapsing solutions. Furthermore, it’s necessary to establish alignment between source and target clusters in scMUSCL. The method learns two distinct sets of clusters: one for the combined source datasets and another for the target dataset. These clusters are optimized separately using cell alignment loss, without direct alignment between them. Consequently, clusters representing the same cell type in different sets may be distant due to distributional differences between source and target datasets. Therefore, a strategy is required to align these clusters across common cell types. The rationale behind this alignment is to encourage the feature extractor to employ the same set of genes to define a target cluster as it did for the corresponding source reference cluster of the same cell type. This alignment process facilitates the transfer of knowledge from annotated source datasets to the unannotated target dataset.

We introduce a novel loss term, termed cluster alignment loss, which focuses on minimizing entropy. We operate under the assumption that our pre-training and initialization of cluster representatives have already positioned similar target and source clusters in close proximity within the feature space. Our goal is to reinforce this proximity while simultaneously increasing the distances between inter-cluster pairs among both source and target clusters. This concept is formalized by quantifying the entropy of pairwise similarities among cluster representatives. Specifically, we anticipate that a target cluster should exhibit high similarity with a corresponding source cluster (if they represent the same cell type), and low similarity with other source clusters and unrelated target clusters. Conversely, each source cluster should demonstrate low similarity with other source clusters. These expectations collectively aim to maintain a low entropy of pairwise cluster similarities.

Cluster alignment loss is illustrated in Fig. 2b. In order to compute it we first define $F\in R^{2n\times d}$ as $F=[C_{1}^{s},\ldots ,C_{n}^{s},C_{1}^{t},\ldots ,C_{n}^{t}]$. Having F defined, we define similarity matrix P such that for each value $p_{i,j}$ in row i and column j we have:

(5) $$p_{i,j}=\frac{\;\text{exp}(\mathrm{sim}(F_{i},F_{j})/\tau )}{Z_{i}},Z_{i}=\sum_{j=1}^{2n}\;\text{exp}\;(\mathrm{sim}(F_{i},F_{j})/\tau ).$$

Here $\mathrm{sim}(F_{i},F_{j})$ is defined as below:

$$\mathrm{sim}(F_{i},F_{j})=\{\begin{array}{cc} 0 & \;i,j\leq n\;or\;i,j\geq n\\ \frac{1}{\mathrm{dist}(F_{i},F_{j})+\epsilon } & \;\mathrm{otherwise} \end{array}.$$

ϵ is a very small number added for numerical stability, and τ controls the distribution concentration degree (Hinton *et al.* 2015) which we set to 1 in our experiments. Now we can define our cluster alignment loss term as:

(6) $$L_{\mathrm{cluster}}=\frac{1}{2n}\sum_{i=1}^{2n}H(P_{i}),$$

where $H(P_{i})$ is the entropy of row i in P, and n is the number of clusters in $C^{s}$ and $C^{t}$. By zeroing $p_{i,j}$ for i,j≤n or i,j>n we are zeroing out the similarity among source clusters and the similarity among target clusters respectively, which means cluster alignment term will align two clusters only if they come from different sets.

### 2.4 scMUSCL loss function

Having all the loss terms defined, the final loss function for the source and the target datasets is defined as follows:

(7) $$L=\sum_{l=1}^{\left|S\right|}L_{\mathrm{cell}}^{s_{l}}+L_{\mathrm{cell}}^{t}+L_{\mathrm{cluster}}.$$

We use loss function L to optimize the feature extractor parameters and cluster representatives in an alternating approach.

## 3 Experimental results

We performed a series of experiments to assess the performance of scMUSCL and compare it with five baseline methods. Among our baseline methods, MARS (Brbić *et al.* 2020) and scArches (Lotfollahi *et al.* 2022) are the most relevant to our work as both are transfer learning methods that exploits multiple annotated source datasets. Two unsupervised methods based on contrastive learning, Contrastive-sc (Ciortan and Defrance 2021) and scNAME (Wan *et al.* 2022) were included in the comparison, as this allows us to ask whether exploiting labeled source datasets in a transfer learning paradigm is beneficial. scDeepCluster (Tian *et al.* 2019) is also chosen as a baseline method since it is a deep-learning-based model that similar to scMUSCL learns feature representations and clustering simultaneously. The last baseline, SIMLR (Wang *et al.* 2018), is a method for learning cell representations, particularly for clustering scRNA-seq data. We chose SIMLR as one of the baselines because it has been widely used in the literature. In all experiments of this section, we used a feature extractor with two hidden layers with 1024 and 256 neurons, where the output layer has 100 neurons (d=100). The reported performance results are the mean results of three independent experiments. The hyper-parameters of each experiment are shown in Supplementary Table S1. For SIMLR we used a Python implementation available in https://github.com/bowang87/SIMLR_PY. For the remaining, we used their official implementations and default settings available on the corresponding GitHub page.

### 3.1 Performance metrics

We used Accuracy and Adjusted Rand Index (ARI) as our performance metrics. The clustering accuracy of different methods is computed after running the Hungarian Maximum Matching Algorithm (Weber and Robinson 2016) on the confusion matrix to find the best mapping between cell-types and cluster labels. Rand Index (RI) is a measure of similarity between two clusterings. It considers all pairs of samples and counts pairs that are assigned in the same or in different clusters in the predicted and true clusterings. For example, true positive (TP) is defined as the number of sample pairs that belong to the same gold standard cluster and the same discovered (by the algorithm) cluster. Adjusted Rand Index is a form of the Rand Index which is adjusted for the chance grouping of elements. We used scikit-learn (https://scikit-learn.org/) metrics API to compute these metrics.

(8) $$\text{RI}=\frac{TP+TN}{TP+FP+FN+TN},$$

 

(9) $$\text{ARI}=\frac{RI-E_{\mathrm{model}}[RI]}{\max(RI)-E_{\mathrm{model}}(RI)}.$$

### 3.2 Datasets

We used 20 real-world datasets to run our experiments. Human and mouse pancreas and kidney datasets were retrieved directly from SingleCellNet GitHub repository (https://github.com/pcahan1/singleCellNet), except for the Tabula Muris dataset which we downloaded directly from its official web page (https://tabula-muris.ds.czbiohub.org). We downloaded the human Peripheral Blood Mononuclear Cells (PBMCs) scRNA-seq data from the SeuratData package (Ding *et al.* 2019). These data consist of seven batches from seven different sequencing platforms: cel-seq2, chrom-v3, indrop, smart-seq, chrom-v2, drop-seq, seq-well. We also used the dataset of six human endoderm-derived organs–lung, esophagus, liver, stomach, small intestine, and colon (Yu *et al.* 2021). A list of these datasets and the details of our pre-processing is available in the Supplementary Material.

### 3.3 Cross-platform knowledge transfer: scMUSCL effectively addresses batch effects across different sequencing platforms

**Figure 4.**
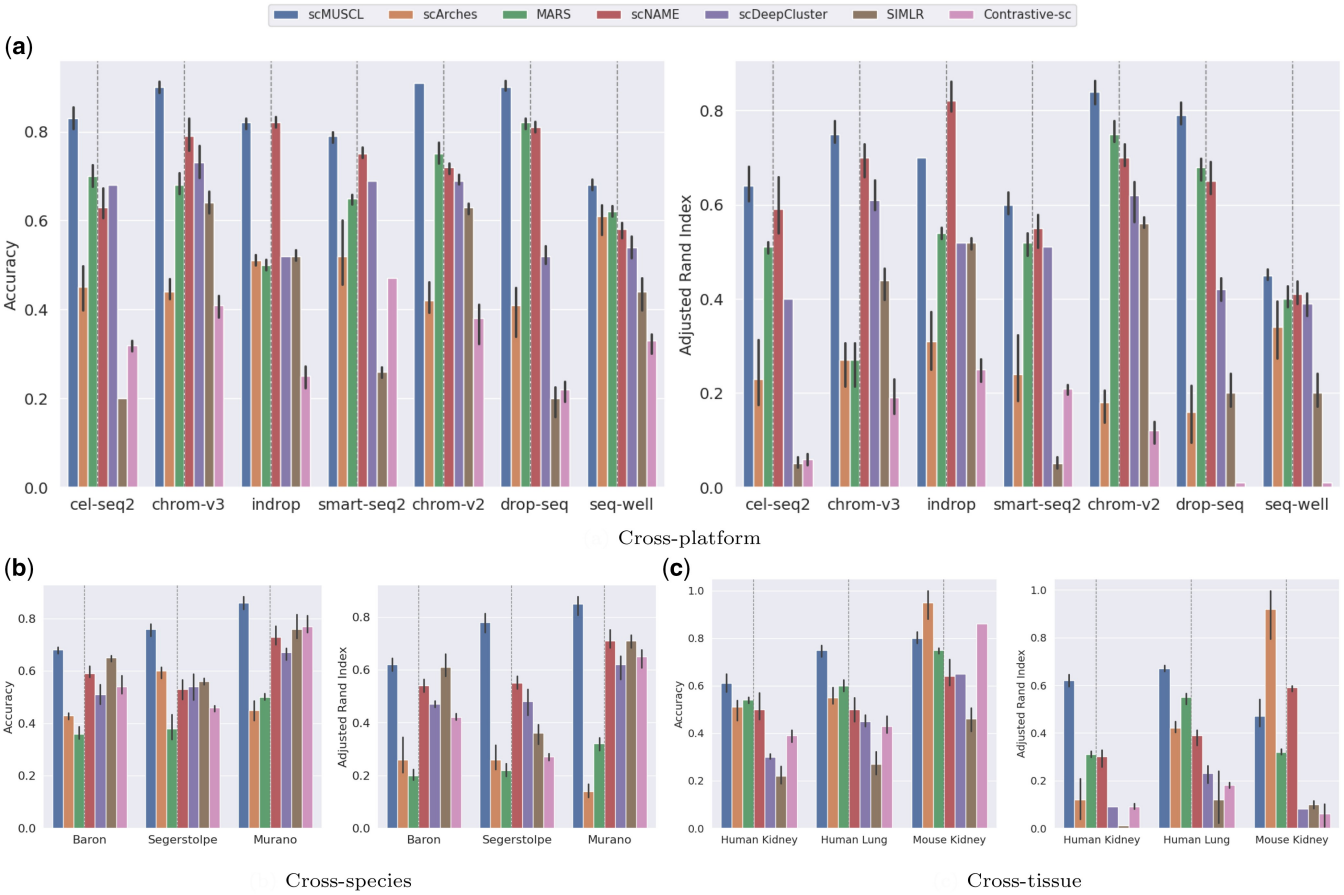
Accuracy and ARI of (a) cross-platform, (b) cross-species, and (c) cross-tissue experiments. The dotted line separates the transfer learning methods and unsupervised methods. (a) For cross-platform experiments, we used seven PBMC datasets. In each experiment, we used one as the target dataset and exploited the rest as the source datasets. (b) In all cross-species experiments, we used two mouse pancreas scRNA-seq data (Baron and Tabula Muris) as the source datasets and one human pancreas as the target dataset. (c) For cross-tissue experiments, we (i) exploited two human pancreas scRNA-seq datasets as the source dataset and one human kidney as the target dataset, (ii) one human esophagus and human short intestine as the source and a human lung as the target, and (iii) two mouse pancreas as the source and a mouse kidney as the target dataset.

scRNA-seq data generated in different laboratories using different library preparation protocols and platforms often suffer from technical artifacts. Collectively, these batch effects can mask the biological information and limit the use of the available data. We first compared the performance of scMUSCL and baseline methods in their ability to transfer knowledge in the presence of batch effect across different sequencing technologies. For this, we used PBMC scRNA-seq data containing seven batches from seven different sequencing platforms: cel-seq2, chrom-v3, indrop, smart-seq, chrom-v2, drop-seq, seq-well. For each experiment, we assigned one dataset as the target dataset and the remaining six datasets served as the source datasets, creating seven different experiments in total. In six out of seven experiments, scMUSCL achieved higher accuracy and ARI than all baseline methods (Fig. 4a, Supplementary Tables S8 and S9). Remarkably, scMUSCL outperformed the second-best method in every experiment, achieving an average improvement of 11.28% in accuracy and 9.89% in ARI. Notably, although scMUSCL, scArches, and MARS all utilize a transfer learning paradigm, scMUSCL consistently demonstrated higher accuracy and ARI compared to both scArches and MARS. This performance gap becomes even more pronounced for chrom-v3 and chrom-v2, the two most widely used platforms in scRNA sequencing.

### 3.4 Cross-species knowledge transfer: scMUSCL effectively transfers knowledge across species

After confirming that scMUSCL effectively overcomes batch effects, we explored its ability to transfer knowledge across species. To evaluate cross-species clustering efficiency, we measured the silhouette coefficient using human pancreas tissue (Murano) as the unannotated target dataset. Without a training dataset, the baseline silhouette coefficient was 0.03. When a single mouse pancreas dataset (Baron) was used for training, the coefficient improved to 0.76. Adding a second mouse dataset (Tabula Muris) yielded a modest further increase, indicating that scMUSCL effectively transfers knowledge across species and can achieve strong clustering performance with limited training data. TSNE plots are available in Figure 3.

Next, we evaluated scMUSCL’s cross-species transfer effectiveness using accuracy and ARI as metrics. With Baron and Tabula Muris as training datasets and three independent human pancreas datasets as targets, scMUSCL consistently outperformed all baselines in both ARI and accuracy (Fig. 4b, Supplementary Table S10). Remarkably, scMUSCL exceeded the second-best model by an average of 14.32% in accuracy and 21.6% in ARI. Notably, MARS performed poorly due to its inability to align source and target clusters under domain shift, leading to domain-discriminative features and negative transfer (Fig. 1).

Interestingly, unsupervised methods performed comparably in cross-species experiments, reflecting the inherent challenges of transferring knowledge across species. Fundamental biological differences, such as gene expression patterns, regulatory networks, and evolutionary divergence, introduce significant discrepancies between source and target datasets, complicating generalization. Despite comparable performance, unsupervised methods have a critical limitation: they require the correct number of clusters in the target dataset—a parameter often unavailable in practical scenarios—highlighting their drawback in real-world applications.

### 3.5 Cross-tissue knowledge transfer: scMUSCL effectively transfers knowledge across different human tissues

Next, we investigated whether scMUSCL can effectively transfer knowledge across different human tissues. To test this, we trained the model on two datasets from human esophagus and small intestine tissues, and evaluated its performance on a target dataset from human lung tissue (see Fig. 4c and Supplementary Table S11). scMUSCL demonstrated a 36.3% higher accuracy and a 59.5% higher ARI compared to MARS, the second-best performing baseline method. To consider an even more challenging case, we used two human pancreas datasets with approximately 7600 and 1900 cells as the source datasets, and a large human kidney dataset with more than 41 000 cells as the target data. We see that even though scMUSCL has relatively small source datasets available to learn from, it still outperforms the second-best performing method by 18%. Next, we analyzed scMUSCL’s performance in transferring knowledge between different mouse tissues. Using two mouse pancreas datasets (Baron and Tabula Muris) as the source and a mouse kidney dataset (Tabula Muris) as the target, we found that MARS outperformed scMUSCL in both accuracy and ARI. Further investigation is needed to understand the reasons behind scMUSCL’s lower performance in this context.

### 3.6 scMUSCL benefits from an increasing number of source datasets in cross-tissue experiments

**Figure 5.**
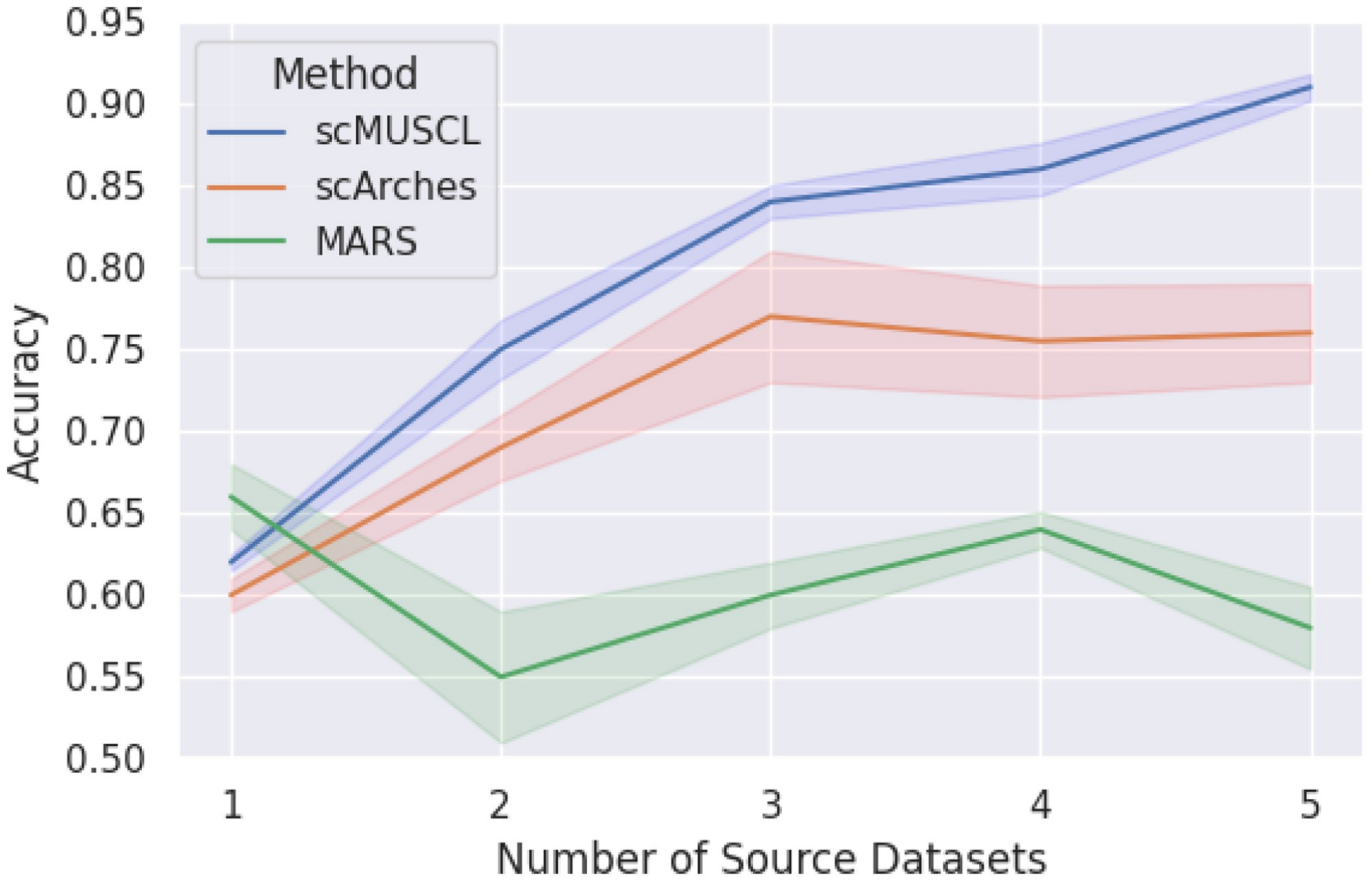
Accuracy of clustering on a human lung tissue with an increasing number of endoderm-derived organs as the source datasets.

To evaluate whether scMUSCL can benefit from multiple source datasets, we performed five more cross-tissue experiments where we increased the number of source datasets by one in each experiment (Fig. 5). To avoid bias in selecting the tissues to add to the training datasets, we selected a set of tissues, where a diverse set of cell types, tissue-specific and shared, are present (Yu *et al.* 2021). We first used esophagus as the only source dataset, and we then added small intestine, colon, stomach, and liver to the source datasets and determined the effect of the number of source datasets in clustering of human lung tissue (Fig. 5). We observed that scMUSCL’s clustering accuracy improves with additional source datasets, rising from 0.62 with one dataset to 0.91 with five. While MARS slightly outperforms scMUSCL with one source dataset, its performance declines as more datasets are added, highlighting its inability to handle multiple domains and batch effects, leading to negative transfer. Similarly, scArches improves with the first three datasets but plateaus afterward, suggesting diminishing returns due to overfitting or limitations in utilizing additional data.

### 3.7 scMUSCL outperforms MARS in estimating the correct number of clusters

Unlike the unsupervised baseline methods, MARS, scArches, and scMUSCL do not require users to specify the number of clusters in the target dataset beforehand. Both methods can automatically estimate the number of clusters during the training process. To evaluate their performance in estimating the correct number of target clusters, we compared their cluster resolution using Root Mean Square Error (RMSE) between the estimated and true number of clusters across all cross-species, cross-platform, and cross-tissue experiments. Our results indicated that scMUSCL had a lower RMSE (Table 1), suggesting that it is more accurate than MARS in estimating the number of clusters in the target dataset.

**Table 1.** Root square mean error (RMSE) between the estimated and the gold standard number of target clusters. The lowest RMSE values, achieved by scMUSCL, are highlighted in bold.

	scMUSCL	scArches	MARS
Cross-species	**2.44**	5.24	5.91
Cross-tissues	**4.47**	8.63	8.06
Cross-platforms	**3.60**	8.90	9.22

### 3.8 Further analysis available in supplementary

We conducted additional analyses of scMUSCL, detailed in the Supplementary Materials. These analyses include evaluating the impact of pre-processing with Harmony on scMUSCL’s performance, assessing its robustness under varying levels of sparsity, examining its runtime, scalability, and memory usage, and performing an ablation study on its individual stages.

## 4 Conclusion

In this article, we addressed the problem of scRNA-seq data clustering, highlighting that most current methods overlook the wealth of available annotated scRNA-seq datasets. To overcome this limitation, we proposed scMUSCL, a multi-source transfer learning method that utilizes multiple annotated scRNA-seq datasets to identify clusters of cells in an unannotated target scRNA-seq dataset. Notably, scMUSCL does not require prior knowledge of the number of target clusters, as it can estimate the correct number during the training process, eliminating the need for additional tools.

We conducted extensive experiments showing that scMUSCL can transfer knowledge across platforms, species, and tissues, significantly outperforming all the baseline scRNA-seq clustering methods. scMUSCL effectively aligns clusters of cell types shared between different source datasets and the target dataset, which we believe is the primary reason it outperforms MARS (Brbić *et al.* 2020) and scArches (Lotfollahi *et al.* 2022), two leading transfer learning-based methods for scRNA-seq clustering.

This article opens several avenues for future work:

1. **Alternative Similarity Measures**: Investigating the impact of using similarity measures other than Euclidean distance such as cosine similarity, Manhattan distance, or Mahalanobis distance could provide insights into their influence on the algorithm.
2. **Robustness to Annotation Errors**: Assess the robustness of the algorithm in terms of its performance when errors are introduced in the original cell type annotation. This could help evaluate its reliability in practical scenarios with noisy or incomplete data.
3. **Weighted Source Datasets**: Currently, scMUSCL assigns equal weights to all source datasets. Future work could focus on adapting the model to assign weights to source datasets based on their relevance to the biological question or their similarity to the target dataset.
4. **Multiple Target Datasets**: Extending the approach to handle multiple target datasets simultaneously could enhance its capacity to model complex cell–cell relationships across diverse contexts.

## Supplementary Material

btaf137_Supplementary_Data

## Data Availability

We used 20 publicly available datasets, including data from SingleCellNet, Tabula Muris, SeuratData, and Yu et al. (2021). Sources for all datasets are listed in the Experimental Results section, with preprocessing details provided in the Supplementary Materials.
