# Supplementary material for: scMUSCL: multi-source transfer learning for clustering scRNA-seq data
Source: Bioinformatics. 2025 Mar 27;41(5):btaf137. doi: 10.1093/bioinformatics/btaf137 (PMC12065430; doi:10.1093/bioinformatics/btaf137)
Supplement: btaf137_Supplementary_Data [file btaf137_supplementary_data.pdf]

scMUSCL: Supplementary Materials

1 Related Works

In this section we review recent machine-learning methods for cell clustering, which we roughly categorize into two groups: unsupervised methods and transfer-learning methods.

1.1 Unsupervised Methods

Earlier works mostly focus on dimensionality reduction, i.e. how to map high dimensional cell profiles into a much lower dimensional space for further downstream tasks such as clustering or visualization, (1; 2; 3; 4; 5). One example is SIMLR (1). SIMLR learns a similarity measure for scRNA-seq data to perform dimension reduction. It combines multiple kernels to learn a distance metric that fits the structure of the data the best. SIMLR also employs a rank constraint in the learned cell-to-cell similarity and graph diffusion to address the challenge of high levels of dropout events. SIMLR couples clustering with the learning of a cell-cell similarity matrix and a respective low-dimensional (latent) representation. SIMLR shows promising results and has been used widely in the literature. In another work, authors employ deep generative neural networks to propose scVI (2). scVI uses stochastic optimization and a variational auto-encoder (6) to aggregate information across similar cells and genes, and to approximate the distributions that underlie the observed expression values. In a more recent deep-neural-networks-based approach, authors propose scDeepCluster (4). scDeepCluster aims to jointly learn feature representations and cell clustering via explicit modelling of scRNA-seq data generation using a denoising autoencoder. Autoencoders are a special kind of neural networks which are suitable for learning a low-dimensional latent representation of high-dimensional data. Denoising autoencoders capture more robust latent representations by learning to predict the original input given the randomly corrupted input. scDeepCluster trains a denoising autoencoder using a zero-inflated negative binomial (ZINB) loss function. To simultaneously learn a clustering of cells as well as feature representations, scDeepCluster employs Kullback-Leibler (KL) divergence on the latent space as described in the 'deep embedded clustering' (DEC) algorithm (7). scziDesk (8) is another clustering method that utilizes denoising autoencoders. scziDesk uses a denoising autoencoder to characterize scRNA-seq data while proposing a soft self-training K-means algorithm to cluster the cell population in the learned latent space. The self-training procedure aggregates similar cells to obtain a more cluster-friendly latent space.

Similar to autoencoders, contrastive learning (9) is a self-supervised method for learning latent representations. Contrastive learning encourages augmentations (views) of the same input to have more similar representations compared to augmentations of different inputs. Contrastive-sc (5) is based on contrastive learning, and it aims to learn cell's latent features that facilitate clustering. In Contrastive-sc, a neural network first learns a representation for each cell through a representation training phase. The representation is then clustered with a general clustering algorithm, such as K-means or Leiden community detection. scNAME (10) is a successor of Contrastive-sc and it incorporates a mask estimation task to a neighbourhood contrastive learning framework for cell representation learning. Since scRNA-seq data is noisy, the mask estimation task helps to reveal uncorrupted data structure and denoise the scRNA-seq data. scNAME also introduces a neighbourhood contrastive paradigm with an offline memory bank, which achieves intra-cluster compactness, yet inter-cluster separation. scNAME shows strong performance and outperforms previous state-of-the-art approaches for single-cell clustering.

1.2 Transfer Learning

All the methods we introduced so far are unsupervised, meaning they only exploit an unannotated scRNA-seq dataset to learn complex intrinsic cell relationships. scmap (11) and Moana (12) are two examples of methods that take advantage of the annotated scRNA-seq datasets as their reference. scmap (11) combines three metrics, Pearson correlation, cosine distance, and Spearman correlation, to quantify the closeness between a cell and the centroids of cell clusters. It projects cells in a target dataset to a lower dimensional space and annotates them based on their correlation with the average cell-type specific gene expression in the source data. Moana (12) is based on support vector machines and uses a linear kernel on PCA-transformed annotated source data to cluster cells in the target data. scAdapt (13) is based on domain adaptation paradigm and it uses adversarial domain adaptation to learn domain-invariant features using annotated source and unannotated target datasets. Domain-invariant features allow one to learn a task (e.g., cell-type annotation) from a source domain and then transfer it to a related target domain. scAdapt is unable to learn from more than one source domain, and it assumes the source and the target datasets follow the same category distribution, meaning it is unable to detect and find unseen cell-types: cell-types that do not exist in the source data but are present in the target data. A more similar appraoch to our work is MARS (14), which is also a multi-source transfer learning method for single-cell clustering that does not require to know the correct number of clusters in advance. MARS considers multiple annotated datasets as the source dataset and one unannotated dataset as the target dataset, and transfers knowledge extracted from the source dataset to cluster cells in the target dataset. The feature extractor in MARS is a stacked auto-encoder and is trained using a k-means-like loss function to map cells to clusters.

2 Datasets and Pre-processing

We used 20 datasets to run our experiments. These datasets are listed in table 2. Please note PBMC dataset by Ding is actually 7 different batches sequenced by 7 different technologies. For each experiment we only kept the genes that are shared among source and target datasets. For Tabula Muris pancreas tissue we removed pancreatic A, D, and PP cell. We also removed 'immune other' from Baron mouse pancreas dataset and renamed 'activated-stellate', and 'quiescent-stellate' to 'stellate' in Baron mouse and human pancreas dataset. We further removed 'unclassified', 'co-expression', 'unclassified endocrine', and 'MHC class II' from Segerstolpe human pancreas dataset and renamed 'PSC' to stellate. In Park human kidney dataset we removed 'ascending loop of Henle', 'novel1', and 'novel2' cells and renamed 'proximal tubule', and 'distal convoluted tubule' to 'tubule'. We also renamed 'collecting duct intercalated cell', 'collecting duct principal cell', and 'collecting duct transitional cell' to 'collecting duct' and renamed 'endothelial, vascular, and descending loop of Henle' to endothelial. In Tabula Muris pancreas heart tissue we renamed 'smooth muscle cell', and 'cardiac muscle cell' to 'muscle'. Lastly, in six endodermal datasets by Yu et. a., we remove undefined cells, renamed 'T cell/NK cell 1' and 'T cell/NK cell 2' into 'T Cell', renamed 'Mesenchyme subtype 1' to 5 and 'Proliferative mesenchyme' to Mesenchyme, renamed 'PNS glia' and 'PNS neuron' to PNS, and renamed 'Distal lung epithelium', 'Gastrointestinal epithelium', and 'Intestinal epithelium' to Epithelium. We used ScanPy [1] python package to log normalize all datasets with the scale factor of 10000.

3 Hyper-parameter Tuning

Chosen values for hyper-parameters of our method are shown in table 1. We did a grid search over a limited number of options for hyper-parameters in each category of experiments (cross-specie, cross-tissue, and cross-platform). For pre-training and fine-tuning learning rate we searched among [0.005,

[1] https://scanpy.readthedocs.io/en/stable/

Experiment	Pre-training Epochs	Pre-training LR	Training Epochs	Training LR	LR Scheduler
Cross-Species	60	0.0005	200	0.0005	×
Cross-Tissue	40	0.0005	200	0.005	✓
Cross-Platform	40	0.0005	300	0.0005	×

Table 1: Selected Hyper-parameter for our experiments.

Specie	Tissue	Source
Mouse	Pancreas	Baron
Mouse	Pancreas	Tabula Muris
Human	Pancreas	Baron
Human	Pancreas	Segerstolpe
Human	Pancreas	Murano
Human	Kidney	Park
Mouse	Kidney	Tabula Muris
Human	PBMC	Ding
Human	Lung	Yu et. al.
Human	Esophagus	Yu et. al.
Human	Liver	Yu et. al.
Human	Stomach	Yu et. al.
Human	Small Intestine	Yu et. al.
Human	Colon	Yu et. al.

Table 2: List of all 20 scRNA-seq datasets we used in our empirical study. Human PBMC dataset by Ding is actually 7 different batches sequenced by 7 different technologies.

0.001, 0.0005]. For pre-training and fine-tuning epoch number we searched among [60, 80] and [200, 300] respectively and we did no early stopping. For our feature extractor we tried two options. One with a hidden layer of 1024 neurons and one with two hidden layers with 1024 and 256 neurons. For the size of extracted latent representations we considered three options [80, 100, 128]. We also tried using a learning rate scheduler with gamma=0.1 and step size of 50.

4 Further Experiments and Results

4.1 Evaluating the Impact of Pre-processing with Harmony on scMUSCL Performance

scMUSCL is inherently designed to address batch effects during its training process, making it robust to batch variability. To evaluate whether scMUSCL could still benefit from additional batch effect removal techniques as a pre-processing step, we utilized Harmony (?), a widely recognized method for batch effect correction. Specifically, Harmony was applied to remove batch effects from the seven PBMC datasets used in our cross-platform experiments. We then repeated the cross-platform experiments using the preprocessed data. The results of these experiments are summarized in Table 3.

Interestingly, the results indicate that using Harmony as a pre-processing step consistently degrades the performance of scMUSCL across all seven PBMC datasets. A plausible explanation for this observation lies in the methodological differences between Harmony and scMUSCL. Harmony operates on the principal components of the dataset, effectively reducing the dimensionality of the input space before applying batch correction. In contrast, scMUSCL operates directly on the full gene expression input space, leveraging the entire gene expression profile to capture subtle biological signals and variations. By working in the full input space, scMUSCL can potentially utilize more comprehensive information, making it better suited for tasks that require precise resolution of cell states and types.

These findings emphasize the importance of aligning pre-processing strategies with the underlying architecture and capabilities of the model. While Harmony is a powerful tool for batch effect correction,

its dimensionality reduction step may inadvertently discard biologically relevant information, thereby limiting its compatibility with models like scMUSCL that are designed to operate on the full feature space.

	cel-seq2	chrom-v3	indrop	smart-seq2	chrom-v2	drop-seq	seq-well
With Harmony	0.75 ± 0.02	0.78 ± 0.05	0.69 ± 0.01	0.76 ± 0.03	0.87 ± 0.02	0.66 ± 0.07	0.59 ± 0.00
Without Harmony	$\mathbf{0.83 \pm 0.02}$	$\mathbf{0.90 \pm 0.01}$	$\mathbf{0.82 \pm 0.01}$	$\mathbf{0.79 \pm 0.01}$	$\mathbf{0.91 \pm 0.00}$	$\mathbf{0.90 \pm 0.01}$	$\mathbf{0.68 \pm 0.01}$

Table 3: Summary of results from cross-platform experiments on seven PBMC datasets, comparing the performance of scMUSCL with and without the use of Harmony for batch effect correction. Despite the effectiveness of Harmony in removing batch effects, the results show a consistent degradation in scMUSCL's performance when Harmony is applied as a pre-processing step.

4.2 Robustness of scMUSCL Under Varying Levels of Sparsity

Single-cell RNA sequencing (scRNA-seq) data often suffers from sparsity due to the drop-out effect, where some genes are not detected in certain cells, leading to missing data. To evaluate the robustness of scMUSCL under varying levels of sparsity, we took a cross-tissue experiment (the one with two mouse kidney as source datasets and one human kidney as the target dataset) and zeroed out non-zero genes with rates of 10%, 30%, and 50%. We assessed the performance of scMUSCL on these datasets by measuring cluster accuracy in the target domain. We observed that scMUSCL maintained high cluster accuracy across all sparsity levels. Specifically, the accuracy was 58%, 53%, and 51% at drop-out rates of 10%, 30%, and 50%, respectively. These results demonstrate that the proposed method is robust to the sparsity challenges commonly observed in scRNA-seq data. By leveraging contrastive pre-training and fine-tuning, scMUSCL effectively learns meaningful representations even in the presence of significant missing data.

4.3 Runtime, Scalability, and Memory Usage Analysis of scMUSCL

We evaluated the runtime, scalability, and memory usage of scMUSCL using synthetic datasets generated using Splatter (15). This approach provided better control over dataset parameters, ensuring systematic analysis. All experiments were conducted on a machine equipped with an Intel Core i7-13700 CPU and an NVIDIA GeForce RTX 4060Ti GPU.
The key factors influencing runtime and memory usage in scMUSCL are the number of source cells, number of target cells, and number of genes. We systematically varied these parameters across three sets of experiments to comprehensively analyze the computational performance of our method. Below, we discuss the results.

4.3.1 Effect of Source Cell Count

In the first set of experiments, the number of target cells and genes were fixed at 1,000 and 30,000, respectively. The number of source cells was progressively increased to 1,000, 2,000, 5,000, 10,000, and 20,000.

As illustrated in Figure 1a, the runtime of scMUSCL increased linearly with the number of source cells, starting from 166 seconds for 1,000 cells and reaching 1,571 seconds for 20,000 cells. A similar trend was observed in Figure 1b, showing linear growth in memory usage from 8,966 MB to 16,690 MB as the number of source cells increased.

This scalability is expected, as the computation required for aligning source cells to target cells grows proportionally with the size of the source dataset.

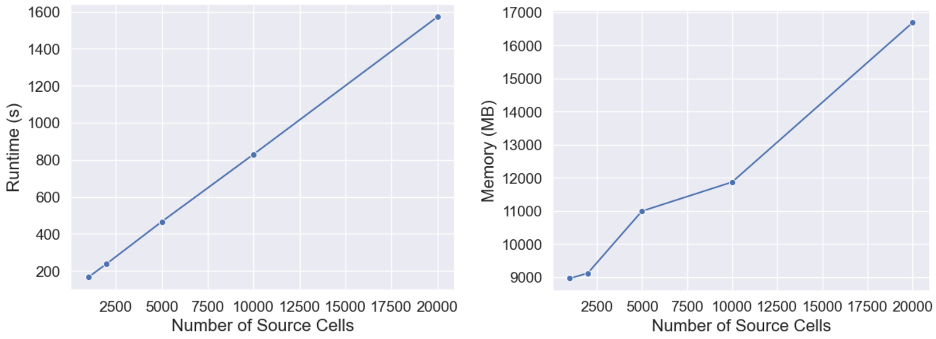

(a) Runtime

(b) Memory Usage

Figure 1: The (a) runtime and (b) memory usage of scMUSCL as a function of the number of source cells. The number of target cells and genes were fixed at 1,000 and 30,000, respectively.

4.3.2 Effect of Target Cell Count

The second set of experiments fixed the number of source cells at 5,000 and the number of genes at 30,000, while the number of target cells was varied: 500, 1,000, 5,000, and 10,000.

Figure 2a demonstrates a linear increase in runtime, ranging from 421 seconds for 500 target cells to 1,143 seconds for 10,000 target cells. Similarly, Figure 2b shows that memory usage scaled linearly, from 11,626 MB for 500 target cells to 16,483 MB for 10,000 target cells.

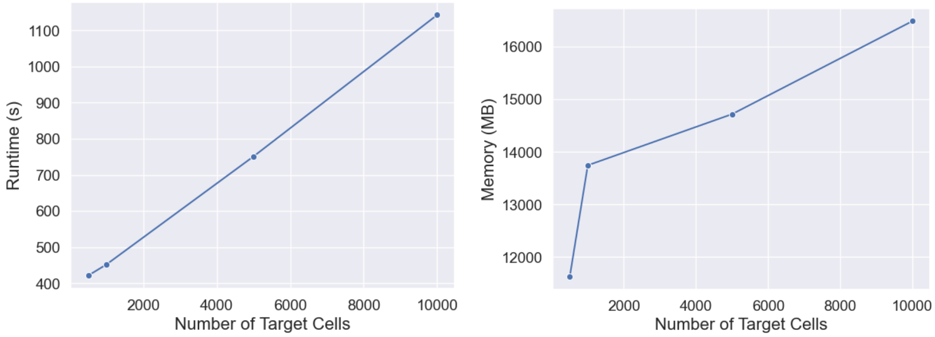

(a) Runtime

(b) Memory Usage

Figure 2: The (a) runtime and (b) memory usage of scMUSCL as a function of the number of target cells. The number of source cells and genes were fixed at 5,000 and 30,000, respectively.

This behavior aligns with the increased computational and memory demand as the number of target cells grows, given the pairwise operations inherent to the method.

4.3.3 Effect of Gene Count

In the final set of experiments, we fixed the number of source cells and target cells at 5,000 and 1,000, respectively, while varying the number of genes: 1,000, 5,000, 10,000, 20,000, and 30,000.

As shown in Figure 3a, the runtime increased linearly, starting at 103 seconds for 1,000 genes and reaching 448 seconds for 30,000 genes. Similarly, memory usage (Figure 3b) increased from 9,686 MB for 1,000 genes to 13,648 MB for 30,000 genes.

This linear scaling indicates that scMUSCL efficiently handles larger feature spaces without exponential increases in computational overhead, making it well-suited for datasets with high-dimensional gene expression profiles.

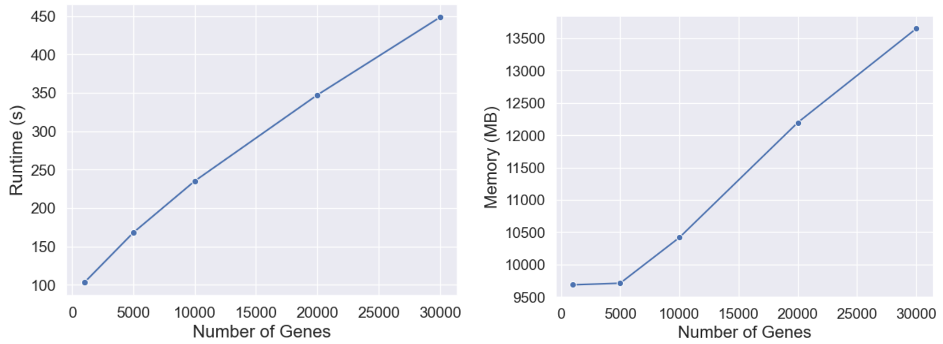

(a) Runtime (b) Memory Usage

Figure 3: The (a) runtime and (b) memory usage of scMUSCL as a function of the number of genes. The number of source cells and target cells were fixed at 5,000 and 1,000, respectively.

Ablation Studies: Cross-Species						
Pancreas Source	Mouse: Baron and Tabula Muris					
Human Target	Baron		Segerstolpe		Murano	
Metric	ACC	ARI	ACC	ARI	ACC	ARI
scMUSCL w/o pre-training	0.46 ± 0.8	0.18 ± 0.12	0.53 ± 0.08	0.14 ± 0.16	0.42 ± 0.05	0.10 ± 0.14
scMUSCL w/o initilization	0.49 ± 0.03	0.41 ± 0.03	0.42 ± 0.02	0.31 ± 0.02	0.52 ± 0.00	0.41 ± 0.00
scMUSCL w/o fine-tuning	0.36 ± 0.00	0.04 ± 0.00	0.52 ± 0.01	0.10 ± 0.02	0.43 ± 0.00	0.07 ± 0.00
scMUSCL	$\mathbf{0.68 \pm 0.00}$	$\mathbf{0.61 \pm 0.03}$	$\mathbf{0.76 \pm 0.07}$	$\mathbf{0.78 \pm 0.12}$	$\mathbf{0.86 \pm 0.02}$	$\mathbf{0.85 \pm 0.05}$

Table 4: Results of the ablation studies. We created three different versions of scMUSCL, removing one stage in each version. Results demonstrate the effectiveness of all three scMUSCL's stages.

4.3.4 Summary of Performance

Across all experiments, scMUSCL demonstrated linear scalability in both runtime and memory usage with respect to the number of source cells, target cells, and genes. These results highlight its efficiency and applicability to large-scale single-cell datasets, a critical requirement for modern bioinformatics workflows.

4.4 Ablation Study on Different Stages of scMUSCL

We created three different versions of scMUSCL to evaluate the effectiveness of each of its three stages: (1) scMUSCL w/o pre-training, in which we did no contrastive pre-training, (2) scMUSCL w/o initialization, in which we used k-means to initialize target clusters, and (3) scMUSCL w/o fine-tuning, in which we did no fine-tuning. As we can see in Table 4, scMUSCL benefits from each of these three stages. Moreover, we plot the accuracy and ARI of the target dataset and the source datasets during training in Figure 4. For this plot, we used a cross-species experiment in which two mouse pancreas tissues from Baron and Tabula Muris are used as the source dataset and Murano human pancreas tissue is used as the target. We see in Figure 4b that our model starts from a high source accuracy, which is because contrastive pre-training has already learned some characteristics of the cells. However, source datasets are not aligned yet. The increasing trend in source accuracy indicates that our fine-tuning stage is aligning source clusters. Looking at Figure 4a we can see the target accuracy rapidly increases at the first few epochs. We believe this is because our model quickly learns to transfer the extracted knowledge from the source datasets to the target dataset. The target accuracy continues increasing as our model learns to align the source and target datasets.

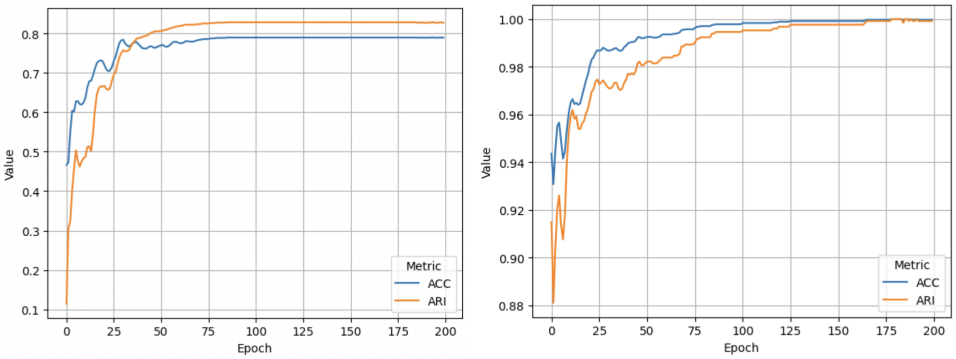

(a) Target Dataset (b) Source Datasets

Figure 4: ARI and ACC of (a) target dataset and (b) source datasets in a cross-species experiment. Here we used two mouse pancreas datasets from Baron and Tabula Muris as source datasets and a human pancreas dataset by Murano as the target dataset.

Tissue	Adj MI	Accuracy	Recall	Adj Rand	NMI	Precision	F1 Score
cel_seq2	0.629 ± 0.015	0.714 ± 0.044	0.785 ± 0.016	0.531 ± 0.051	0.640 ± 0.014	0.798 ± 0.011	0.775 ± 0.010
chrom_v3	0.769 ± 0.028	0.807 ± 0.042	0.861 ± 0.025	0.645 ± 0.057	0.770 ± 0.028	0.865 ± 0.041	0.838 ± 0.042
indrop	0.686 ± 0.009	0.807 ± 0.006	0.840 ± 0.005	0.684 ± 0.012	0.687 ± 0.009	0.661 ± 0.011	0.690 ± 0.007
smart_seq2	0.764 ± 0.016	0.858 ± 0.019	0.898 ± 0.006	0.684 ± 0.047	0.771 ± 0.015	0.909 ± 0.009	0.900 ± 0.009
chrom_v2	0.826 ± 0.009	0.905 ± 0.007	0.939 ± 0.003	0.842 ± 0.013	0.827 ± 0.009	0.796 ± 0.011	0.836 ± 0.009
drop_seq	0.637 ± 0.007	0.721 ± 0.021	0.758 ± 0.015	0.580 ± 0.027	0.638 ± 0.007	0.641 ± 0.013	0.618 ± 0.006
seq_well	0.545 ± 0.011	0.659 ± 0.002	0.759 ± 0.007	0.445 ± 0.003	0.547 ± 0.011	0.558 ± 0.004	0.549 ± 0.004

Table 5: Cross-Platform: comparison of various metrics across different tissues when using supervised contrastive pre-training.

4.5 Supervised Vs. Self-Supervised Contrastive Learning

scMUSCL employs self-supervised contrastive learning, where positive samples are generated through random masking of cells. A known challenge with self-supervised contrastive learning is the potential formation of false negative pairs. Additionally, the masking process can sometimes produce positive pairs with distinct biological characteristics, which may not accurately represent true relationships. To explore whether scMUSCL pre-training could benefit from additional guidance, we conducted a new set of experiments where cell type information from the source experiments was used to generate positive pairs and eliminate false negative pairs. The results, provided in Supplementary Tables 6, 7, and 5, revealed that this approach slightly degraded performance. This finding aligns with existing literature on contrastive learning, which suggests that supervised contrastive learning tends to produce more compact clusters compared to self-supervised contrastive learning. While compact clusters can be beneficial in some cases, they can degrade performance in certain scenarios, such as transfer learning, by reducing generalizability (16).

4.6 Further Results

Tables 10, 11, 9, 8 further show the results of our experiments. Each number is a mean value of three different runs. We also report further metrics for these experiments in Tables 12, 13, and 14.

Target	Adj MI	Accuracy	Recall	Adj Rand	NMI	Precision	F1 Score
Human Pancreas Baron	0.631 ± 0.048	0.545 ± 0.029	0.595 ± 0.020	0.396 ± 0.058	0.633 ± 0.047	0.529 ± 0.037	0.475 ± 0.037
Human Pancreas Segerstolpe	0.638 ± 0.031	0.596 ± 0.039	0.738 ± 0.037	0.417 ± 0.015	0.642 ± 0.031	0.674 ± 0.046	0.625 ± 0.050
Human Pancreas Murano	0.720 ± 0.040	0.659 ± 0.050	0.687 ± 0.055	0.549 ± 0.054	0.723 ± 0.040	0.738 ± 0.083	0.689 ± 0.075

Table 6: Cross-Species: Comparison of various metrics for different pancreas datasets when using supervised contrastive pre-training.

Target	Adj MI	Accuracy	Recall	Adj Rand	NMI	Precision	F1 Score
Human Kidney Park	0.471 ± 0.024	0.871 ± 0.001	0.202 ± 0.019	0.557 ± 0.050	0.471 ± 0.024	0.146 ± 0.026	0.164 ± 0.024
Human Lung Yu	0.521 ± 0.000	0.887 ± 0.000	0.560 ± 0.000	0.314 ± 0.000	0.424 ± 0.000	0.544 ± 0.000	0.551 ± 0.000
Mouse Kidney TM	0.211 ± 0.000	0.887 ± 0.000	0.500 ± 0.000	0.290 ± 0.000	0.104 ± 0.000	0.444 ± 0.000	0.470 ± 0.000

Table 7: Cross-Tissues: comparison of various metrics for human and mouse kidney and lung datasets when using supervised contrastive learning.

Cross-Platform						
Target	chrom-v2		drop-seq		seq-well	
Metric	ACC	ARI	ACC	ARI	ACC	ARI
scNAME	0.72 ± 0.01	0.70 ± 0.02	0.81 ± 0.01	0.65 ± 0.03	0.58 ± 0.02	0.41 ± 0.03
MARS	0.42 ± 0.03	0.18 ± 0.03	0.41 ± 0.09	0.16 ± 0.13	0.61 ± 0.06	0.34 ± 0.08
scDeepCluster	0.69 ± 0.04	0.62 ± 0.07	0.52 ± 0.02	0.42 ± 0.03	0.54 ± 0.02	0.39 ± 0.02
SIMLR	0.63 ± 0.01	0.56 ± 0.01	0.45 ± 0.05	0.20 ± 0.07	0.44 ± 0.03	0.20 ± 0.03
Contrastive-sc	0.38 ± 0.04	0.12 ± 0.02	0.22 ± 0.02	0.00 ± 0.00	0.33 ± 0.02	0.01 ± 0.00
scArches	0.75 ± 0.02	0.75 ± 0.02	0.82 ± 0.01	0.68 ± 0.02	0.62 ± 0.01	0.40 ± 0.02
scMUSCL	**0.91 ± 0.00**	**0.84 ± 0.02**	**0.90 ± 0.01**	**0.79 ± 0.04**	**0.68 ± 0.01**	**0.45 ± 0.01**

Table 8: Results of cross-platform experiments on a PBMC dataset. This data consists of seven batches from seven different sequencing platforms. Here we use one batch as the target datasets and the remaining 6 batches as the source dataset. Reported numbers are mean and standard deviation over three runs.

Cross-Platform								
Target	cel-seq2		chrom-v3		indrop		smart-seq2	
Metric	ACC	ARI	ACC	ARI	ACC	ARI	ACC	ARI
scName	0.63 ± 0.02	0.59 ± 0.11	0.79 ± 0.03	0.70 ± 0.05	**0.82 ± 0.03**	**0.71 ± 0.02**	0.75 ± 0.01	0.55 ± 0.09
MARS	0.45 ± 0.04	0.23 ± 0.08	0.44 ± 0.02	0.27 ± 0.06	0.51 ± 0.01	0.31 ± 0.05	0.52 ± 0.08	0.24 ± 0.12
scDeepCluster	0.68 ± 0.00	0.40 ± 0.00	0.73 ± 0.06	0.61 ± 0.06	0.52 ± 0.00	0.41 ± 0.00	0.69 ± 0.00	0.51 ± 0.00
SIMLR	0.20 ± 0.00	0.05 ± 0.01	0.64 ± 0.04	0.44 ± 0.05	0.52 ± 0.01	0.32 ± 0.00	0.26 ± 0.01	0.05 ± 0.01
Contrastive-sc	0.32 ± 0.01	0.06 ± 0.01	0.41 ± 0.04	0.19 ± 0.05	0.25 ± 0.02	0.07 ± 0.02	0.47 ± 0.00	0.21 ± 0.01
scArches	0.70 ± 0.02	0.51 ± 0.01	0.80 ± 0.03	0.68 ± 0.02	0.55 ± 0.03	0.54 ± 0.01	0.65 ± 0.01	0.52 ± 0.02
scMUSCL	**0.83 ± 0.02**	**0.64 ± 0.04**	**0.90 ± 0.01**	**0.75 ± 0.03**	**0.82 ± 0.01**	0.70 ± 0.00	**0.79 ± 0.01**	**0.60 ± 0.02**

Table 9: Results of cross-platform experiments on a PBMC dataset. This data consists of seven batches from seven different sequencing platforms. Here we use one batch as the target datasets and the remaining 6 batches as the source dataset. Reported numbers are mean and standard deviation over three runs.

Cross-Species						
Pancreas Source	Mouse: Baron and Tabula Muris					
Human Target	Baron		Segerstolpe		Murano	
Metric	ACC	ARI	ACC	ARI	ACC	ARI
scNAME	.0.59 ± 0.02	0.54 ± 0.02	0.53 ± 0.03	0.55 ± 0.02	0.74 ± 0.03	0.71 ± 0.03
MARS	0.43 ± 0.01	0.20 ± 0.07	0.60 ± 0.02	0.26 ± 0.06	0.45 ± 0.03	0.14 ± 0.02
scDeepCluster	0.51 ± 0.03	0.47 ± 0.01	0.54 ± 0.04	0.48 ± 0.04	0.67 ± 0.02	0.62 ± 0.04
SIMLR	0.65 ± 0.01	0.61 ± 0.04	0.56 ± 0.01	0.36 ± 0.03	0.76 ± 0.04	0.71 ± 0.02
Contrastive-sc	0.54 ± 0.03	0.42 ± 0.01	0.46 ± 0.01	0.27 ± 0.01	0.77 ± 0.03	0.65 ± 0.03
scArches	0.36 ± 0.02	0.20 ± 0.02	0.38 ± 0.04	0.22 ± 0.02	0.50 ± 0.01	0.32 ± 0.02
scMUSCL	**0.68 ± 0.01**	**0.62 ± 0.02**	**0.76 ± 0.02**	**0.78 ± 0.05**	**0.86 ± 0.02**	**0.85 ± 0.03**

Table 10: Results of cross-species experiment when using two mouse pancreas tissues as the source datasets and three independent human pancreas tissue as the target dataset. Reported numbers are mean and standard deviation over three runs.

Cross-Tissues						
Source	Human Pancreas: Baron, Segerstolpe		Human Yu: Esophagus, short intestine		Mouse Pancreas: Baron, TM	
Target	Kidney Park Human		Yu Lung Human		Kidney Tabula Muris Mouse	
Metric	ACC	ARI	ACC	ARI	ACC	ARI
scNAME	0.50 ± 0.07	0.30 ± 0.03	0.50 ± 0.04	0.39 ± 0.03	0.64 ± 0.05	0.59 ± 0.01
MARS	0.51 ± 0.05	0.12 ± 0.07	0.55 ± 0.03	0.42 ± 0.02	$\mathbf{0.96 \pm 0.15}$	$\mathbf{0.92 \pm 0.11}$
scDeepCluster	0.30 ± 0.01	0.09 ± 0.00	0.45 ± 0.02	0.23 ± 0.03	0.65 ± 0.00	0.08 ± 0.00
SIMLR	0.22 ± 0.03	0.01 ± 0.00	0.27 ± 0.06	0.12 ± 0.09	0.46 ± 0.04	0.10 ± 0.02
Contrastive-sc	0.39 ± 0.02	0.09 ± 0.01	0.43 ± 0.04	0.18 ± 0.01	0.86 ± 0.00	0.06 ± 0.04
scArches	0.54 ± 0.01	0.31 ± 0.01	0.60 ± 0.02	0.55 ± 0.02	0.75 ± 0.01	0.32 ± 0.01
scMUSCL	$\mathbf{0.61 \pm 0.05}$	$\mathbf{0.33 \pm 0.05}$	$\mathbf{0.75 \pm 0.02}$	$\mathbf{0.67 \pm 0.01}$	0.80 ± 0.02	0.47 ± 0.05

Table 11: Results of three cross-tissue experiments. In the first experiment, we used two human pancreas tissues as source datasets and transfer the extracted knowledge to find clusters in a human kidney dataset. In the second experiment we learned from human esophagus and small intestine tissues and used a human lung tissue as the target dataset. In the third experiment we learned from two mouse pancreas datasets to cluster a human kidney dataset. Reported numbers are mean and standard deviation over three runs.

Cross-Species					
target	adj-mi	recall	nmi	precision	f1-score
Human Pancreas Baron	0.748 ± 0.021	0.622 ± 0.059	0.749 ± 0.021	0.619 ± 0.019	0.566 ± 0.020
Human Pancreas Segerstolpe	0.832 ± 0.037	0.720 ± 0.057	0.834 ± 0.037	0.761 ± 0.066	0.722 ± 0.059
Human Pancreas Murano	0.825 ± 0.022	0.687 ± 0.035	0.827 ± 0.021	0.672 ± 0.051	0.672 ± 0.041

Table 12: Further metrics for cross-species experiments. Reported numbers are mean and standard deviation over three runs.

Cross-Tissue					
target	adj-mi	recall	nmi	precision	f1-score
Human Kidney Park	0.505 ± 0.040	0.456 ± 0.055	0.505 ± 0.040	0.442 ± 0.026	0.419 ± 0.038
Human Lung Yu	0.683 ± 0.016	0.656 ± 0.021	0.684 ± 0.009	0.659 ± 0.019	0.619 ± 0.021
Mouse Kidney TM	0.489 ± 0.080	0.807 ± 0.056	0.500 ± 0.078	0.980 ± 0.028	0.879 ± 0.022

Table 13: Further metrics for cross-tissue experiments. Reported numbers are mean and standard deviation over three runs.

Cross-Platform					
target	adj-mi	recall	nmi	precision	f1-score
cel-seq2	0.718 ± 0.016	0.829 ± 0.022	0.725 ± 0.016	0.844 ± 0.010	0.827 ± 0.010
chrom-v3	0.831 ± 0.010	0.921 ± 0.004	0.832 ± 0.010	0.889 ± 0.001	0.899 ± 0.003
indrop	0.735 ± 0.004	0.889 ± 0.004	0.736 ± 0.004	0.753 ± 0.009	0.781 ± 0.010
smart-seq2	0.745 ± 0.012	0.867 ± 0.010	0.752 ± 0.012	0.865 ± 0.008	0.843 ± 0.012
chrom-v2	0.851 ± 0.009	0.942 ± 0.002	0.852 ± 0.009	0.875 ± 0.012	0.897 ± 0.007
drop-seq	0.782 ± 0.014	0.900 ± 0.011	0.783 ± 0.014	0.820 ± 0.011	0.827 ± 0.011
seq-well	0.552 ± 0.014	0.817 ± 0.010	0.554 ± 0.014	0.739 ± 0.010	0.729 ± 0.011

Table 14: Further metrics for cross-platform experiments. Reported numbers are mean and standard deviation over three runs.

References

[1] Wang, B. *et al.* Simlr: A tool for large-scale genomic analyses by multi-kernel learning. *Proteomics* **18**, 1700232 (2018).

[2] Lopez, R., Regier, J., Cole, M. B., Jordan, M. I. & Yosef, N. Deep generative modeling for single-cell transcriptomics. *Nature methods* **15**, 1053–1058 (2018).

[3] Stuart, T. *et al.* Comprehensive integration of single-cell data. *Cell* **177**, 1888–1902 (2019).

[4] Tian, T., Wan, J., Song, Q. & Wei, Z. Clustering single-cell rna-seq data with a model-based deep learning approach. *Nature Machine Intelligence* **1**, 191–198 (2019).

[5] Ciortan, M. & Defrance, M. Contrastive self-supervised clustering of scrna-seq data. *BMC bioinformatics* **22**, 1–27 (2021).

[6] Kingma, D. P. & Welling, M. Auto-encoding variational bayes. *arXiv preprint arXiv:1312.6114* (2013).

[7] Xie, J., Girshick, R. & Farhadi, A. Unsupervised deep embedding for clustering analysis. In *International conference on machine learning*, 478–487 (PMLR, 2016).

[8] Chen, L., Wang, W., Zhai, Y. & Deng, M. Deep soft k-means clustering with self-training for single-cell rna sequence data. *NAR genomics and bioinformatics* **2**, lqaa039 (2020).

[9] Chen, T., Kornblith, S., Norouzi, M. & Hinton, G. A simple framework for contrastive learning of visual representations. In *International conference on machine learning*, 1597–1607 (PMLR, 2020).

[10] Wan, H., Chen, L. & Deng, M. scname: neighborhood contrastive clustering with ancillary mask estimation for scrna-seq data. *Bioinformatics* **38**, 1575–1583 (2023).

[11] Kiselev, V. Y., Yiu, A. & Hemberg, M. scmap: projection of single-cell rna-seq data across data sets. *Nature methods* **15**, 359–362 (2018).

[12] Wagner, F. & Yanai, I. Moana: a robust and scalable cell type classification framework for single-cell rna-seq data. *BioRxiv* 456129 (2018).

[13] Zhou, X. *et al.* scadapt: Virtual adversarial domain adaptation network for single cell rna-seq data classification across platforms and species. *bioRxiv* (2021).

[14] Brbić, M. *et al.* Mars: discovering novel cell types across heterogeneous single-cell experiments. *Nature methods* **17**, 1200–1206 (2020).

[15] Zappia, L., Phipson, B. & Oshlack, A. Splatter: simulation of single-cell rna sequencing data. *Genome biology* **18**, 1–15 (2017).

[16] Islam, A. *et al.* A broad study on the transferability of visual representations with contrastive learning. In *Proceedings of the IEEE/CVF International Conference on Computer Vision*, 8845–8855 (2021).
